# Shear Wave Predictions of Achilles Tendon Loading during Human Walking

**DOI:** 10.1038/s41598-019-49063-7

**Published:** 2019-09-17

**Authors:** Emily M. Keuler, Isaac F. Loegering, Jack A. Martin, Joshua D. Roth, Darryl G. Thelen

**Affiliations:** 1 Department of Mechanical Engineering, University of Wisconsin-Madison, Madison, WI 53706 USA; 2 Department of Biomedical Engineering, University of Wisconsin-Madison, Madison, WI 53706 USA; 3 Department of Materials Science and Engineering, University of Wisconsin-Madison, Madison, WI 53706 USA

**Keywords:** Biomedical engineering, Mechanical engineering

## Abstract

The evaluation of *in vivo* muscle-tendon loads is fundamental to understanding the actuation of normal and pathological human walking. However, conventional techniques for measuring muscle-tendon loads in the human body are too invasive for use in gait analysis. Here, we demonstrate the use of noninvasive measures of shear wave propagation as a proxy for Achilles tendon loading during walking. Twelve healthy young adults performed isometric ankle plantarflexion on a dynamometer. Achilles tendon wave speed, tendon moment arms, tendon cross-sectional area and ankle torque were measured. We first showed that the linear relationship between tendon stress and wave speed squared can be calibrated from isometric tasks. There was no significant effect of knee angle, ankle angle or loading rate on the subject-specific calibrations. Calibrated shear wave tensiometers were used to estimate Achilles tendon loading when walking at speeds ranging from 1 to 2 m/s. Peak tendon stresses during pushoff increased from 41 to 48 MPa as walking speed was increased, and were comparable to estimates from inverse dynamics. The tensiometers also detected Achilles tendon loading of 4 to 7 MPa in late swing. Late swing tendon loading was not discernible in the inverse dynamics estimates, but did coincide with passive stretch of the gastrocnemius muscle-tendon units. This study demonstrates the capacity to use calibrated shear wave tensiometers to evaluate tendon loading in locomotor tasks. Such technology could prove beneficial for identifying the muscle actions that underlie subject-specific movement patterns.

## Introduction

Motion analysis labs have generated a wealth of data describing the kinematics and kinetics of walking in healthy populations. These normative data provide a basis for identifying abnormalities in gait patterns that arise from pathologies such as stroke, cerebral palsy and osteoarthritis. Clinical labs commonly perform comparisons of individual and normative gait patterns to evaluate the cause of gait disorders and to plan treatments. For example in patients with cerebral palsy, such treatments can include spasticity medications, dorsal rhizotomies and orthopedic surgical procedures^1,2^, all of which are intended to alter muscle actions and thereby correct gait abnormalities. With this in mind, it is disconcerting that there remain no feasible means to measure muscle actions when evaluating gait. Traditional motion analysis can characterize loading at the joint level, but resolving the underlying soft tissue loads requires complex models and assumptions regarding muscular coordination^3^. Direct measurement of muscle-tendon loads via implantable sensors^4,5^ is possible, but not practical for use in clinical gait analysis. Noninvasive sensors based on sound wave transmission have been introduced, but sound wave speed is primarily dependent on tissue elasticity rather than loading^6^. Hence, there remains a need for noninvasive sensors of muscle-tendon loads that are suitable for analyzing gait in a clinical setting.

Our lab has recently introduced shear wave tensiometry as a noninvasive approach for gauging *in vivo* muscle-tendon loads during movement^7^. The tensiometers accomplish this by tracking shear wave propagation speed in tendon as a proxy for axial loading. The fundamental basis of this technology is the recognition that squared tendon wave speed varies in proportion to axial stress in tendinous tissue^7^. The constant of proportionality depends on the effective density of the tendon, which includes both the tendon tissue density and added mass due to entrained motion of adjacent tissues and fluid^8^. It is not yet known how to estimate effective density for intact tendons. An alternative is to empirically calibrate shear wave tensiometers under simple conditions that allow for tendon stress to be estimated from external force measurements. Thereafter, tendon wave speeds could be used to predict tendon loads during complex movements, such as walking. Before applying such an approach, it is important to evaluate whether tensiometer calibrations are robust over the range of postures and loading conditions that can arise in movement.

In this study, we implemented an empirical approach for calibrating the Achilles tendon stress-wave speed relationship and applied it to walking. The first objective was to assess consistency of the calibration across postures, loading rates and subjects. The second objective was to use calibrated tensiometers to predict Achilles tendon stress during walking and to compare the results to load estimates based on traditional motion analysis. This calibration approach and normative data provide a basis for assessing Achilles tendon loading in individuals exhibiting gait disorders.

## Methods

Twelve healthy young adults (6 females, 6 males, mean (standard deviation) age: 23.6 (2.8) years, height: 1.75 (0.12) m, mass: 72.5 (11.9) kg) participated in this study. Subjects had no history of lower limb fractures, ligament, or tendon injury in the prior 6 months. There were no significant differences in age between the males and females, though males were taller (p < 0.001) and had greater mass (p < 0.0001). All subjects provided informed consent under an IRB protocol approved by the Health Sciences Institutional Review Board of the University of Wisconsin-Madison. All experiments were performed in accordance with relevant guidelines and regulations. Subjects underwent isometric testing, MR imaging and moment arm measurement to calibrate the tensiometers, and then performed a set of walking tasks to evaluate tensiometer performance (Supplemental Fig. 1).

### Shear wave tensiometry

A shear wave tensiometer was used to monitor Achilles tendon shear wave speed throughout testing. The tensiometer consisted of a piezoelectric-actuated (PK4JQP2, Thorlabs, Inc.) tapping device and two single-axis miniature accelerometers (Model 352C23, PCB Piezotronics) mounted in series against the skin superficial to the tendon^7^. The tapping device was powered by an open-loop piezo controller (MDT694B, Thorlabs, Inc.) driven via a 50 Hz square wave. Accelerometers were secured in 3D-printed housings that were embedded in a silicone mold that maintained a 10 mm spacing between the two accelerometers. The tensiometer was positioned over the Achilles tendon, with the distal accelerometer positioned 35 ± 8 mm proximal to the superior aspect of the calcaneus. Accelerometer signals were amplified (Model 480B21, PCB Piezotronics) and sampled at 50 kHz via a data acquisition system (USB-6363, National Instruments).

**Figure 1:**
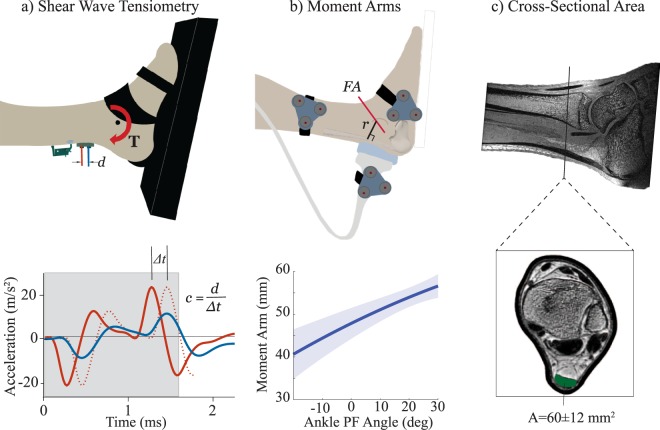
(**a**) Subjects performed cyclic isometric exertions while two accelerometers measured the skin motion associated with an induced shear wave propagating in the tendon. Cross-correlation of the signals within an adaptive window (see gray box that includes acceleration peaks induced by the tap event) was used to determine the propagation time *Δt*, and hence wave speed *c*. (**b**) Coupled ultrasound and motion analysis collections were used to characterize the Achilles tendon moment arm, *r*, as a function of ankle plantarflexion (PF) rotation about a functional axis (*FA*)^13^. (**c**) MR images were segmented to compute the Achilles tendon cross-sectional area, *A*, at the location where the accelerometer array was placed.

Shear wave speeds were calculated by computing the time delay, *Δt*, between the arrival of the wave at the first and second accelerometers (Fig. 1a). The time delay was calculated by finding the time delay that maximized the normalized cross-correlation between the two accelerometer signals over a time window after the tap event. The duration of the cross-correlation window was set to include the transient acceleration peaks induced by a tap event (Fig. 1a). Sub-sample estimation of the time delay was found using a local 3-point cosine fit of the normalized cross-correlation values^9^. For each tap, the shear wave speed, *c*, was computed as *c* = *d/Δt*, where *d* was the fixed inter-accelerometer distance (10 mm).

### Dynamometry

Subjects were positioned in an isokinetic dynamometer (System 4, Biodex Medical Systems, Inc.) with their ankle strapped to a foot plate and their tibiotalar joint aligned with the dynamometer rotation axis. Each subject performed cyclic (0.5 Hz) isometric exertions between relaxed and moderate effort at six postures: 3 ankle plantarflexion angles (−10, 0 and 10 deg) at 2 knee flexion angles (20 and 90 deg). In the 20 deg knee flexion-0 deg ankle posture, subjects repeated the isometric exertions at rates of 0.25, 0.5 and 1.0 Hz. Cyclic exertion rate was maintained via an audible cue provided by a metronome. Applied ankle torque and ankle angle were monitored at 50 kHz in synchrony with the tensiometer data throughout the testing.

### Walking

Subjects completed ten walking trials, with two 10-second trials at speeds of 1.0, 1.25, 1.5, 1.75 and 2.0 m/s on an instrumented split-belt treadmill (Bertec, Corp.). A motion capture system (Motion Analysis Corp.) recorded the three-dimensional trajectories of markers placed on the subjects’ pelvis, thigh, shank and foot at 200 Hz. Ground reaction forces were recorded at 2000 Hz. Standard inverse kinematics and inverse dynamics techniques were used to compute the lower extremity kinematics and kinetics throughout the walking trials^10^. Wireless surface EMG sensors (Trigno, Delsys Inc.) were secured over the right medial gastrocnemius, soleus, and tibialis anterior muscles. EMG signals were recorded at 2000 Hz, full-wave rectified and then bi-directionally low-pass filtered at 20 Hz using a third order Butterworth filter. Heel strike events were identified from the vertical ground reaction force and used to extract individual gait cycles from each walking trial.

### Achilles tendon moment arms

Subjects were positioned prone with their right foot extended over the edge of an examination table. Marker triads were fixed over the lateral aspects of the midfoot, midshank and on the side of a 60 mm linear array ultrasound transducer (L14-5/60, BK Medical). The transducer was manually positioned longitudinally over the subject’s Achilles Tendon (Fig. 1b). The foot was strapped to a rigid plate that was used to slowly rotate the foot from maximum dorsiflexion to maximum plantarflexion. We simultaneously recorded the kinematics (Optotrak Certus, Northern Digital Inc) of marker clusters affixed to the shank, foot and transducer. Kinematics were recorded at 100 Hz in synchrony with cine ultrasound B-mode images (SonixTOUCH Research, BK Medical), which were recorded at 19 frames per second. Kinematic data was linearly interpolated to match the timing of ultrasound image frames. The transducer marker cluster was used to transform ultrasound images into the shank reference frame.

We manually identified the superficial and deep edges of the Achilles tendon in each B-mode image and defined the Achilles tendon line of action as the best fit line midway between the two edges. Marker trajectories were bi-directionally low-pass filtered using a Butterworth filter with a cut-off frequency of 6 Hz. At each frame, singular value decomposition was used to compute the homogeneous transformation between the shank and foot from the positions of the cluster markers on the respective segments^11^. A functional axis (*FA*) was then computed as the best-fit screw axis that described the foot motion with respect to the shank^12^. The Achilles tendon moment arm at each frame was computed as the perpendicular distance between the tendon line of action and the *FA*^13^. A quadratic fit of the moment arms relative to ankle angle was used to estimate the moment arm, *r*, at each angle observed during isometric and walking trials.

### Achilles tendon cross-sectional area

Subjects were positioned supine in a 3 T scanner (Signa PET/MR, GE Healthcare). A GEM Medium Flex Array Coil was secured around the ankle. The ankle was imaged using a three-dimensional spoiled gradient recall-echo sequence that used iterative decomposition with echo asymmetry and least squares estimation for fat-water separation (IDEAL-SPGR)^14^. Three dimensional images were collected (in-plane axial resolution, 0.39 × 0.39 mm; slice thickness, 0.5 mm; matrix, 512 × 512 × 76; flip angle, 14°). We located the transverse image plane associated with the measured position of the tensiometer proximal to the calcaneus. We then manually segmented the tendon in the in-phase images and computed the cross-sectional area, *A* (Fig. 1c).

### Tensiometer calibration

For the isometric exertions, Achilles tendon stress, *σ*, was computed assuming the ankle torque, *T*, was generated by plantarflexor muscle forces transmitted through the Achilles tendon and that antagonist muscles generated no force, i.e. *σ* = *T*/*rA*. A tensioned beam model predicts that tendon stress varies in proportion to squared wave speed under physiological loading conditions^7^. Hence, least squares parameter estimation was used to estimate the gain, *β*, and offset, *α*, that best describe the linear relationship between tendon stress and squared wave speed (Eq. 1):

1 $${\rm{\sigma }}=\beta {c}^{2}+\alpha $$

**Figure 2:**
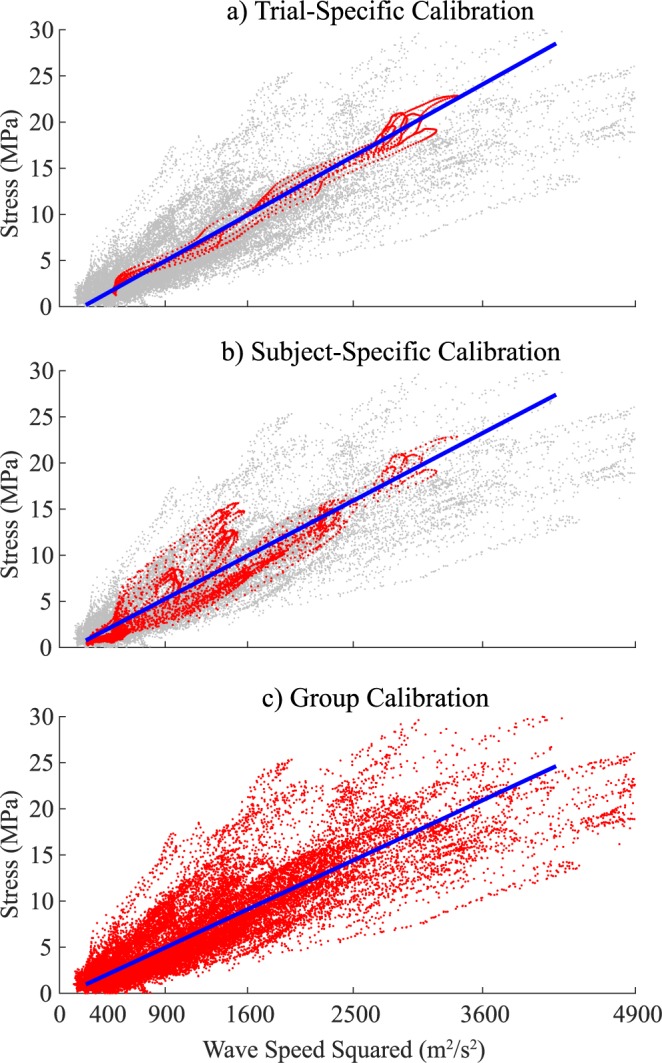
Least squares parameter estimation of the gain between squared wave speed and stress. (**a**) Trial-specific calibrations (top) were performed using data collected at a single posture and loading rate. (**b**) Subject-specific calibrations (middle) were performed by including data from all postures and loading rates tested for a given subject. (**c**) Group calibration (bottom) was performed by including all wave speed and stress data from isometric tasks performed by all subjects. Red symbols represent data used for the three calibration approaches.

We performed tensiometer calibrations three ways (Fig. 2):

1. Trial-specific – parameter estimation was performed on a per-trial basis using the wave speed and stress data collected at a given posture and loading rate,
2. Subject-specific – parameter estimation was performed after pooling the wave speed and stress data from all 8 isometric tasks that a subject performed, and
3. Group – parameter estimation was performed after pooling the wave speed and stress data from all trials of all subjects.

For each case, the tensiometer prediction error was determined by computing the root mean-squared (RMS) error between the tendon stress obtained from ankle torque and that predicted from the measured wave speed. We also computed the coefficient of determination, *R*^2^, to ascertain how much of the variance in stress was described by the calibrated tensiometer.

### Tendon stress during walking

Tendon stress, *σ*_*sw*_, was predicted from shear wave speed during walking using both the subject-specific and group calibration gains. For each subject, we assumed that the minimum shear wave speed, *c*_*min*_, collected over all walking trials represented a zero-load state, such that stress could be computed as (Eq. 2):

2 $${{\rm{\sigma }}}_{sw}=\beta ({c}^{2}-{c}_{min}^{2})$$

We separately used the ankle plantarflexor torque computed via inverse dynamics, *T*_*id*_, to estimate the Achilles tendon stress, *σ*_*id*_ (Eq. 3):

3 $${\sigma }_{id}=\{\begin{array}{ll}\frac{{T}_{id}}{Ar(\theta )} & {T}_{id} > 0\\ 0 & {T}_{id}\le 0\end{array}$$

As in the isometric case, we assumed that plantarflexion torques were generated completely by the triceps surae and that antagonist muscles generated no force. In this equation, the Achilles tendon moment arm, *r(θ*), was estimated from the subject-specific fits of tendon moment arm vs. ankle angle, *θ*. We assumed that inverse dynamics tendon stress was zero when ankle dorsiflexion torques (*T*_*id*_ < 0) were present.

### Statistical analyses

A two-way repeated measures analysis of variance (ANOVA) was performed to investigate the effects of posture (2 knee angles, 3 ankle angles) on the gain, *β*, computed in the trial-specific calibrations. A one-way repeated measures ANOVA was performed to investigate the dependence of the gain on loading rate. Statistical significance was set at *p*  < *0.05*. We assessed the wave speed-stress calibration by computing the RMS error and coefficient of determination between tendon stress estimated from wave speed and tendon stress estimated from dynamometer ankle torque. For the walking trials, we characterized the RMS difference, the precision (standard deviation of difference) and the bias (mean difference) between the shear wave and inverse dynamics estimates of tendon stress. We also computed the coefficient of determination, which was the variance of non-zero inverse dynamics stress, *σ*_*id*_, that was explained by the shear wave speed stress, *σ*_*sw*_. Finally, one-way repeated measures ANOVA were used to investigate the effects of walking speed on peak tendon wave speed, stress and normalized force during both the stance and swing phases of walking.

## Results

Achilles tendon stress and squared wave speed were highly correlated for all isometric tasks, with mean coefficients of determination (*R*^2^) of 0.98 to 0.99 across subjects (Table 1). The calibration gain, *β*, estimated for isometric tasks ranged from 6.8 to 8.0 *kPa·s*^2^/*m*^2^, with no significant variations attributable to ankle angle, knee angle or loading rate. Trial-specific calibrations produced RMS stress prediction errors ranging from 0.7 to 1.7 MPa across the isometric tasks, with an overall average of 1.0 MPa. Subject-specific calibrations, obtained by a single fit to all isometric data for a subject, reduced the average *R*^2^ to 0.96 and increased the RMS stress prediction errors to 1.9 MPa, a 90% increase from the trial-specific case (Table 2). A group calibration, obtained by fitting data from all subjects, further reduced *R*^2^ to 0.88 and increased average RMS stress prediction errors to 2.9 MPa.

**Table 1:** Mean (s.d.) calibration gains β, root-mean squared (RMS) errors and coefficients of determination R^2^ obtained via calibration performed on shear wave speed and tendon stress data from isometric trials. There were no significant effects of knee flexion angle, ankle plantarflexion angle or loading rate on estimates of the gain β.

Knee, deg	Ankle, deg	Rate, Hz	β, kPa·s^2^/m^2^	RMS Error, MPa	R^2^
20	−10	0.50	8.0 (3.0)	1.7 (1.3)	0.98 (0.02)
20	0	0.50	7.7 (2.4)	1.0 (0.6)	0.99 (0.02)
20	10	0.50	7.6 (2.1)	0.8 (0.5)	0.98 (0.01)
90	−10	0.50	7.6 (4.3)	1.1 (0.6)	0.99 (0.01)
90	0	0.50	6.8 (2.5)	1.0 (0.6)	0.99 (0.01)
90	10	0.50	7.7 (2.8)	0.7 (0.3)	0.99 (0.01)
20	0	0.25	7.3 (2.4)	0.9 (0.6)	0.99 (0.02)
20	0	1.00	7.4 (2.5)	1.0 (0.5)	0.98 (0.02)

**Table 2:** Mean (s.d.) calibration gains (β), root-mean squared (RMS) errors and coefficients of determination (R^2^) obtained via calibration performed on trial-specific data, subject-specific data and on pooled data across all subjects in the group.

	Trial-Specific	Subject-Specific	Group
β (kPa·s^2^/m^2^)	7.5 (2.7)	6.9 (2.3)	6.0
RMS Error (MPa)	1.0 (0.7)	1.9 (1.0)	2.9 (1.5)
R^2^	0.98 (0.02)	0.96 (0.02)	0.88 (0.08)

**Figure 3:**
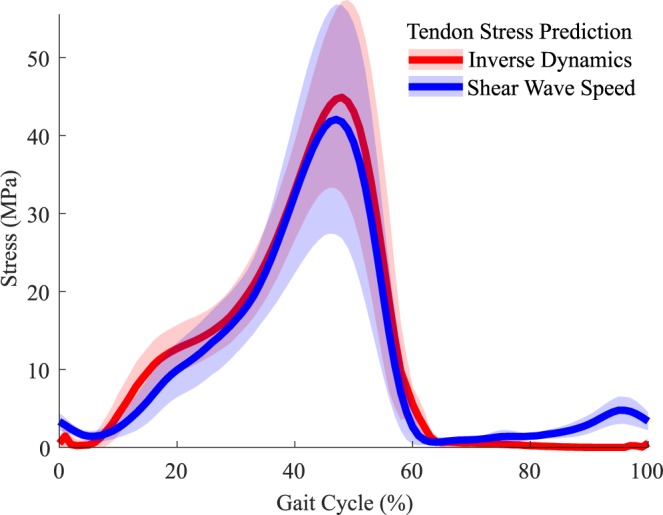
Ensemble average (±1 s.d.) Achilles tendon stress estimates over a gait cycle at the 1.5 m/s walking speed. There is close correspondence between inverse dynamics and shear wave predictions of tendon stress throughout stance. During late swing, shear wave speed detects tendon loading that is not evident in the inverse dynamics data.

There was good temporal agreement between stress predicted by wave speed and inverse dynamics throughout the stance phase of walking (Fig. 3). Differences in the predicted stress patterns did emerge during swing. Shear wave data revealed terminal swing tendon loading that was not evident in the inverse dynamics data. Average RMS differences in stress predictions during stance ranged from 7.5 to 8.6 MPa, representing 17.2–18.3% of the peak stress (Table 3). These differences increased when using group calibration parameters, with average RMS differences of 9.3 to 9.6 MPa (18.9–22.4%).

**Table 3:** Mean (s.d.) bias, precision, root-mean squared (RMS) difference and coefficient of determination (R^2^) between shear wave and inverse dynamics estimates of Achilles tendon stress during stance across a range of walking speeds.

Calibration Data	Walking Speed (m/s)				
	1.0	1.25	1.5	1.75	2.0
**Subject-Specific**					
Bias (MPa)	−1.9 (6.0)	−1.6 (6.1)	−2.0 (6.4)	−1.9 (6.4)	−1.6 (6.1)
Precision (MPa)	5.1 (2.5)	5.6 (2.4)	5.9 (2.8)	6.3 (2.6)	6.2 (2.6)
RMS Diff (MPa)	7.5 (3.6)	7.9 (3.3)	8.4 (3.7)	8.6 (3.7)	8.3 (3.5)
RMS Diff (% peak)	18.3 (9.5)	18.1 (7.8)	17.8 (7.5)	17.6 (6.8)	17.2 (6.9)
R^2^	0.87 (0.12)	0.87 (0.10)	0.87 (0.10)	0.88 (0.07)	0.87 (0.08)
**Group**					
Bias (MPa)	−2.9 (8.5)	−2.6 (8.2)	−3.1 (8.1)	−3.2 (8.3)	−2.7 (7.6)
Precision (MPa)	6.1 (3.8)	6.4 (3.4)	6.4 (3.7)	6.5 (3.8)	6.8 (3.2)
RMS Diff (MPa)	9.4 (6.2)	9.6 (5.5)	9.3 (6.1)	9.3 (6.7)	9.3 (5.5)
RMS Diff (% peak)	22.4 (12.7)	21.6 (10.9)	19.7 (10.9)	18.9 (11.5)	19.0 (9.8)
R^2^	0.80 (0.19)	0.81 (0.16)	0.83 (0.17)	0.84 (0.17)	0.83 (0.16)

Predictions of stress during walking reached a peak of 41 MPa during push off at the slowest (1.0 m/s) walking speed. Peak stress in stance significantly (p < 0.05) increased with walking speed, reaching 48 MPa (+17%) at the 2.0 m/s speed (Table 4). The corresponding tendon force estimates ranged from 3.41 to 3.95 times body weight.

**Table 4:** Mean (s.d.) peak wave speed, tendon stress, and normalized force significantly increased with walking speed during both stance and swing phase. (*p < 0.05, **p < 0.005).

Speed, m/s	Wave Speed, m/s		Stress, MPa		Force, BW	
	Stance**	Swing**	Stance*	Swing**	Stance*	Swing**
1.00	79.4 (18.5)	27.1 (4.2)	40.7 (14.5)	4.1 (1.5)	3.41 (1.24)	0.34 (0.13)
1.25	81.7 (17.0)	28.0 (4.5)	43.0 (13.9)	4.5 (1.9)	3.59 (1.12)	0.38 (0.16)
1.50	83.1 (15.5)	29.6 (4.9)	44.9 (15.1)	5.0 (1.9)	3.73 (1.14)	0.43 (0.17)
1.75	84.6 (16.8)	31.9 (5.8)	46.8 (17.3)	6.1 (2.7)	3.86 (1.25)	0.52 (0.24)
2.00	85.6 (17.5)	34.1 (5.6)	47.9 (17.5)	7.0 (2.7)	3.95 (1.25)	0.59 (0.24)

Achilles tendon stress during swing phase consistently peaked at ~95% of the gait cycle, coinciding closely with terminal knee extension. Average swing phase stress increased with walking speed, ranging from 4.1 MPa at the slowest (1.0 m/s) walking speed to 7.0 MPa at the fastest (2.0 m/s) speed (Table 4). These stresses corresponded to peak tendon loads of 0.34 and 0.59 times body weight, respectively.

## Discussion

This study investigated the calibration of shear wave tensiometers and their use for assessing absolute Achilles tendon loads during walking. A simple linear model was sufficient for capturing the relationship between squared wave speed and tendon stress during isometric exertions. Calibrations did not vary significantly across a range of limb postures and loading rates, suggesting that it is viable to use a single task for calibration. Shear wave predictions of tendon stress significantly increased with walking speed, reaching ~4 times body weight at the fastest speed. Notably, the tensiometers also detected passive tendon loading in late swing that were not discernable in joint level kinetics. These results set the stage for using shear wave tensiometry to assess subject-specific tendon loading patterns in walking, which could be useful for evaluating gait disorders and planning treatments.

The linear relationship between squared wave speed and stress is consistent with a tensioned beam model of tendon^7^. The constant of proportionality, or gain, is dependent on the effective density of the tissue. In this study, we estimated an average subject-specific calibration gain of *6.9 kPa*/*m*^2^/*s*^2^ (*or 6900* *kg*/*m*^3^). This value is considerably higher than the actual tissue density (~1120 kg/m^3^)^15^. This difference is, in part, attributable to the added mass effects associated with the tendon entraining motion in adjacent tissues. Indeed we have shown in *ex vivo* studies that tendons exhibit shear wave speeds that are 20% lower in water than in air, likely due to the entrained motion of water adjacent to the tendon^8^. We are currently performing *in situ* cadaveric studies with adjacent tissues intact and dissected to better understand the morphological factors that determine effective density.

There was no significant effect of knee angle, ankle angle or loading rate on the estimated calibration parameters. However, stress prediction errors were considerably larger (+90%) when using a collection of eight isometric tasks than when calibrating to a single task. While these variations were not systematic with posture, it is possible that underlying tendon motion could alter the cross-sectional area, and hence stress, of the tendon underlying the tensiometer. Given the lack of a significant postural effect, our results suggest a single task should be sufficient to calibrate a tensiometer for the Achilles tendon of an individual. Thereafter, the calibrated tensiometer could be used to evaluate tendon loading during arbitrary movements. It would be sensible to choose a posture that corresponds with greater joint torque capacity to enable calibration over a broad range of tendon stress. There is clearly some subject-specificity in the wave speed-stress relationship, with a 34% coefficient of variation in the calibration gain, *β*, across subjects (Table 1). Likewise, use of a single calibration for all subjects resulted in isometric stress prediction errors that were 53% greater than achieved via subject-specific calibration. Thus, it would seem desirable to perform a subject-specific calibration if one is interested in absolute tendon loads.

This study demonstrates the unique potential to use tensiometers to assess absolute tendon loading during walking. Our prior study revealed strong agreement between wave speed and joint torque patterns during walking, but did not perform the calibrations needed to transform the wave speed data into tissue load measures. A comparison of wave speed-predicted tendon stresses to traditional inverse dynamics estimates provides a perspective on the errors one might expect in using the sensors for estimating tissue loads. The independently computed stress trajectories were highly correlated (average *R*^2^ > *0.87*), with average RMS differences ranging from 17.2 to 18.3% of peak stress values (Table 3). Using a nominal group calibration produced RMS differences that were from 12–25% greater than those obtained with subject-specific calibrations (Table 3). Note that variability in marker placement and center of pressure estimation can lead to error in inverse dynamics torque estimates. Hence, the difference in stress estimates during walking are likely the result of errors both in the tensiometer calibration and the inverse dynamics measurements.

The magnitude of shear wave-predicted stress during walking agree well with prior estimates obtained via invasive sensors and motion analysis. Finni *et al*. estimated peak Achilles tendon stress of 21 MPa during walking at speeds of 1.1 to 1.8 m/s using optic fiber techniques^5^. Komi *et al*. reported peaks stresses of 59 MPa using a buckle transducer^4,16^ across a similar walking speed range. Our stress estimates during walking fall between the invasive sensor estimates. A prior motion analysis study reported average ankle torques that increased from 112 to 127 Nm across the 1.0 to 2.0 m/s walking speeds considered here^17^. These torques would correspond to Achilles tendon stresses ranging from 44 to 50 MPa, assuming average tendon moment arms and cross-sectional areas observed here. Our shear wave-based estimates of tendon stress (average range of 41–48 MPa) are comparable to these values. However, our tendon load measures were ascertained directly from a small tensiometer secured over the skin, a methodology considerably simpler than using treadmill motion analysis lab and inverse biomechanical models to estimate internal tissue loads^3^. Further, it is possible to use the shear wave tensiometers outside a laboratory environment. This capability permits an array of opportunities in rehabilitation, sports and ergonomics to assess muscle-tendon loading during movements performed in natural environments.

**Figure 4:**
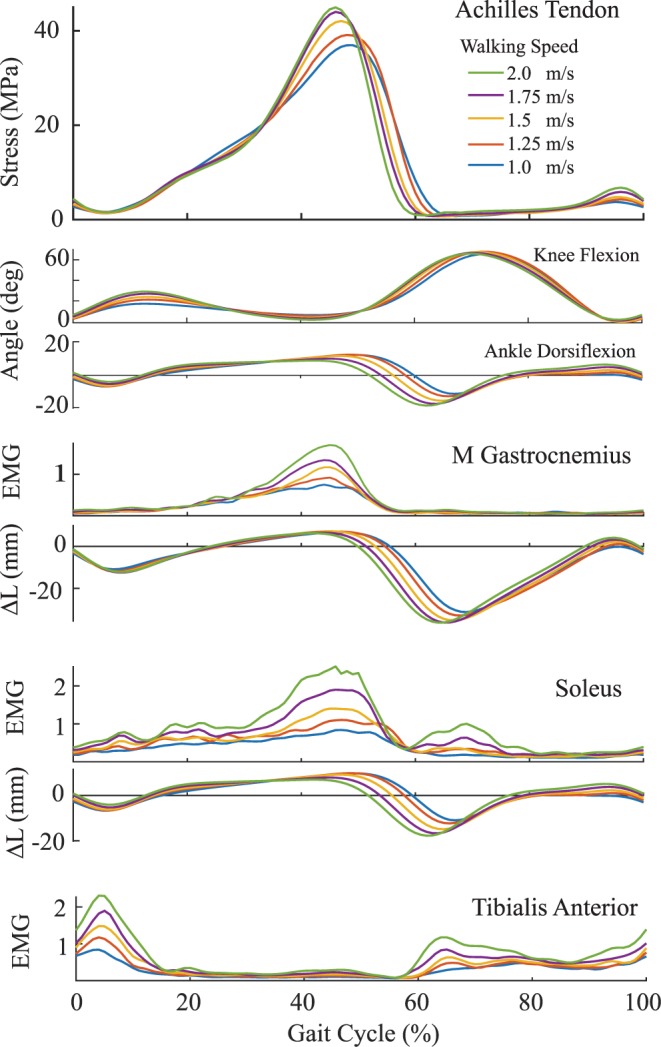
Ensemble Achilles tendon stress patterns at the five walking speeds. Peak stress and both medial gastrocnemius and soleus activity progressively increase with walking speed. Late swing Achilles tendon loading arises without discernable plantarflexor muscle activity. However, gastrocnemius elongation (*ΔL*, relative to upright length) in late swing aligns temporally with tendon loading, suggesting the loading is induced by passive stretch. Simultaneous tibialis anterior muscle activity counters the ankle torque induced by the Achilles tendon.

One of the more interesting observations of this study was the characteristic Achilles tendon loading patterns seen in the late swing phase of walking. Peak swing phase tendon loads ranged from 34% to 59% body weight, increasing significantly with speed. Both the gastrocnemius and soleus muscles were relatively inactive in late swing (Fig. 4), such that the tendon force must have been induced passively. To investigate this further, we used the joint kinematics data to estimate the medial gastrocnemius and soleus muscle-tendon kinematics throughout the gait cycle. Ankle angle was coupled with our moment arm data to assess muscle-tendon excursions about the ankle. A 20 mm knee flexor moment arm^18^ was used to estimate gastrocnemius excursions resulting from knee motion. These calculations reveal a close relationship between peak tendon force and gastrocnemius muscle-tendon length in late swing (Fig. 4), with both measures increasing with walking speed. This observation suggests that late swing tendon force is likely attributable to passive stretch of the biarticular gastrocnemius. This passive Achilles tendon force is countered by activation of the dorsiflexors (Fig. 4). Such a mechanism could serve as an effective means of storing energy from limb deceleration in late swing and stiffening the limb prior to heel strike. The capacity to detect passive tissue loading is particularly relevant when translating the tensiometers for use in evaluating gait disorders. For example, it is recognized that abnormal triceps surae actions can contribute to equinus walking in children with Cerebral Palsy^19^. However, it can be challenging to distinguish between static contractures and dynamic triceps surae tightness, which is important when planning treatment^19^. The capacity of the tensiometer to detect both active and passive loading could provide a mechanism for delineating the underlying condition.

It may prove challenging for subjects with gait disorders to perform isometric exertions in a manner suitable for tensiometer calibration. For example, difficulties with selective motor control could induce coordination patterns (e.g. co-contraction) that make it challenging to infer tendon stress from net joint torque. Our data would suggest that in these cases a generic calibration may prove suitable and could generate tendon load estimates that are on par in accuracy with joint torque data.

Clearly, calibrating an *in vivo* tissue load sensor is a challenge given that a ground-truth measure of loading is not obtainable. Our *in vivo* tendon stress estimates relied on measurements of tendon size and moment arm and assumptions regarding muscle load sharing. We adopted a coupled ultrasonic imaging and motion capture technique to characterize the tendon moment arms during passive ankle rotation^20^. There is some evidence of moment arm enhancement with loading^21^, which would not have been accounted for by our technique. Further, we computed an average stress assuming the tendon loading was uniform over the cross-sectional area of the relaxed tissue. This assumption ignores the reduction in tendon cross-section that occurs with tension^22^, and hence results in slightly lower stress estimates than might actually occur. Finally, our estimates of tendon stress ignore other plantarflexors such as tibialis posterior and peroneus longus. Musculoskeletal models suggest that muscles other than the plantarflexors contribute less than 15% to maximum plantarflexor torque^23^. Including the other muscles would inherently reduce the calibration gain attributed to the Achilles tendon and hence the tendon stresses estimated during walking.

The tensiometers were positioned at consistent locations relative to the calcaneus. However, this resulted in the tensiometer being positioned over the free Achilles tendon in 8 subjects and over the soleus aponeurosis in 4 subjects. This distinction is attributable to the individual variability in soleus muscle-tendon junction that has been reported previously^24^. It is possible that these differences in anatomical placement ultimately contributed to variability in the calibration gains across subjects. This study focused on calibrating and evaluating the performance of shear wave tensiometers to track Achilles tendon loading during walking. We previously demonstrated the potential to track shear wave speeds in the patellar and hamstring tendons during running. Additional work is needed to asses whether the calibration results and predictive performance extend to these other tendons and movements.

In summary, we have demonstrated that shear wave tensiometers can be calibrated using simple isometric experimental paradigms. The tensiometers were shown to produce viable estimates of Achilles tendon stress across a range of walking speeds. Hence, shear wave tensiometry provides an exciting new approach for evaluating absolute tissue loading patterns during normal gait, and could provide a means to investigate muscle-tendon actions underlying gait disorders.

## Supplementary information

Supplementary Figure

## Data Availability

Datasets generated during the current study are available from the corresponding author on request.
