# Supplementary material for: Shear Wave Predictions of Achilles Tendon Loading during Human Walking
Source: Sci Rep. 2019 Sep 17;9:13419. doi: 10.1038/s41598-019-49063-7 (PMC6748912; doi:10.1038/s41598-019-49063-7)
Supplement: Supplementary file 1 — Supplementary Figure [file 41598_2019_49063_MOESM1_ESM.pdf]

Shear Wave Predictions of Achilles Tendon Loading during Human Walking

Emily M. Keuler, Isaac Loegering, Jack A. Martin, Joshua D. Roth, Darryl G. Thelen

University of Wisconsin-Madison

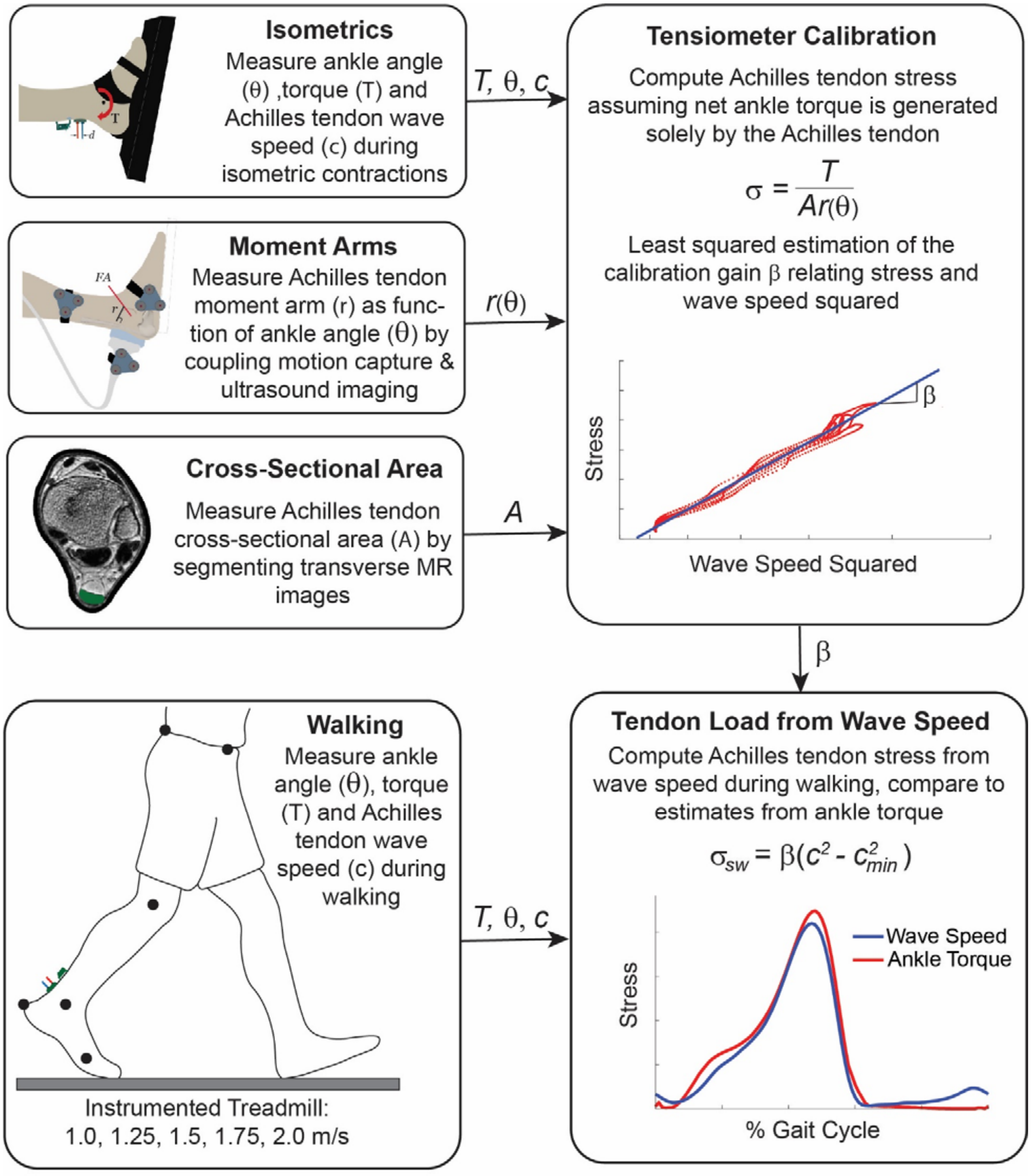

Supplementary Figure 1 | Schematic of the subject-specific measurements, tensiometer calibration and use of calibrated tensiometers to predict tendon loads during walking.
